# A Practical Approach to Language Complexity: A Wikipedia Case Study

**DOI:** 10.1371/journal.pone.0048386

**Published:** 2012-11-07

**Authors:** Taha Yasseri, András Kornai, János Kertész

**Affiliations:** 1 Department of Theoretical Physics, Budapest University of Technology and Economics, Budapest, Hungary; 2 Computer and Automation Research Institute, Hungarian Academy of Sciences, Budapest, Hungary; 3 Center for Network Science, Central European University, Budapest, Hungary; Max Planck Institute for the Physics of Complex Systems, Germany

## Abstract

In this paper we present statistical analysis of English texts from Wikipedia. We try to address the issue of language complexity empirically by comparing the simple English Wikipedia (Simple) to comparable samples of the main English Wikipedia (Main). Simple is supposed to use a more simplified language with a limited vocabulary, and editors are explicitly requested to follow this guideline, yet in practice the vocabulary richness of both samples are at the same level. Detailed analysis of longer units (n-grams of words and part of speech tags) shows that the language of Simple is less complex than that of Main primarily due to the use of shorter sentences, as opposed to drastically simplified syntax or vocabulary. Comparing the two language varieties by the Gunning readability index supports this conclusion. We also report on the topical dependence of language complexity, that is, that the language is more advanced in conceptual articles compared to person-based (biographical) and object-based articles. Finally, we investigate the relation between conflict and language complexity by analyzing the content of the talk pages associated to controversial and peacefully developing articles, concluding that controversy has the effect of reducing language complexity.

## Introduction

Readability is one of the central issues of language complexity and applied linguistics in general [1]. Despite the long history of investigations on readability measurement, and significant effort to introduce computational criteria to model and evaluate the complexity of text in the sense of readability, a conclusive and fully representative scheme is still missing [2]–[4]. In recent years the large amount of machine readable user generated text available on the web has offered new possibilities to address many classic questions of psycholinguistics. Recent studies, based on text-mining of blogs [5], web pages [6], online forums 7,8, etc, have advanced our understanding of natural languages considerably.

Among all the potential online corpora, Wikipedia, a multilingual online encyclopedia [9], which is written collaboratively by volunteers around the world, has a special position. Since Wikipedia content is produced collaboratively, it is a uniquely unbiased sample. As Wikipedias exist in many languages, we can carry out a wide range of cross-linguistic studies. Moreover, the broad studies on social aspects of Wikipedia and its communities of users [10]–[18] makes it possible to develop sociolinguistic descriptions for the linguistic observations.

One of the particularly interesting editions of Wikipedia is the *Simple English Wikipedia* [19] (Simple). Simple aims at providing an encyclopedia for people with only basic knowledge of English, in particular children, adults with learning difficulties, and people learning English as a second language. See Table 1 comparing the articles for ‘April’ in Simple and Main. In this work, we reconsider the issue of language complexity based on the statistical analysis of a corpus extracted from Simple. We compare basic measures of readability across Simple and the standard English Wikipedia (Main) [20] to understand how simple is Simple in comparison. Since there are no supervising editors involved in the process of writing Wikipedia articles, both Simple and Main are uncorrected (natural) output of the human language generation ability. The text of Wikipedias is emerging from contributions of a large number of independent editors, therefore all different types of personalization and bias are eliminated, making it possible to address the fundamental concepts regardless of marginal phenomena.

**Table 1:** The articles on *April* in Main English and Simple English Wikipedias.

Main	Simple
April is the fourth month of the year in the Julian and Gregorian calendars, and one of four months with a length of 30 days. The traditional etymology is from the Latin aperire, “to open,” in allusion to its being the season when trees and flowers begin to “open”.	April is the fourth month of the year. It has 30 days. The name April comes from that Latin word aperire which means “to open”. This probably refers to growing plants in spring.

Readability studies on different corpora have a long history; see [21] for a summary. In a recent study [22], readability of articles published in the *Annals of Internal Medicine* before and after the reviewing process is investigated, and a slight improvement in readability upon the review process is reported. Wikipedia is widely used to extract concepts, relations, facts and descriptions by applying natural language processing techniques [23]. In [24]–[27] different authors have tried to extract semantic knowledge from Wikipedia aiming at measuring semantic relatedness, lexical analysis and text classification. Wikipedia is used to establish topical indexing methods in [28]. Tan and Fuchun performed query segmentation by combining generative language models and Wikipedia information [29]. In a novel approach, Tyers and Pienaarused used Wikipedia to extract bilingual word pairs from interlingual hyperlinks connecting articles from different language editions [30]. And more practically, Sharoff and Hartley have been seeking for “suitable texts for language learners”, developing a new complexity measure, based on both lexical and grammatical features [31]. Comparisons between Simple and Main for the selected set of articles show that in most cases Simple has less complexity, but there exist exceptional articles, which are more readable in Main than in Simple. In a complementary study [32], Simple is examined by measuring the Flesch reading score [33]. They found that Simple is not simple enough compared to other English texts, but there is a positive trend for the whole Wikipedia to become more readable as time goes by, and that the tagging of those articles that need more simplifications by editors is crucial for this achievement. In a new class of applications [34]–[36], Simple is used to establish automated text simplification algorithms.

## Methods

We built our own corpora from the dumps [37] of Simple and Main Wikipedias released at the end of 2010 using the WikiExtractor developed at the University of Pisa Multimedia Lab (see Text S2 for the availability of this and other software packages and corpora used in this work). The Simple corpus covers the whole text of Simple Wikipedia articles (no talk pages, categories and templates). For the Main English Wikipedia, first we made a big single text including all articles, and then created a corpus comparable to Simple by randomly selecting texts having the same sizes as the Simple articles. In both samples HTML entities were converted to characters, MediaWiki tags and commands were discarded, but the anchor texts were kept.

Simple uses significantly shorter words (4.68 characters/word) than Main (5.01 characters/word). We can define ‘same size’ by equal number of characters (see Condition CB in Table 2), or by equal number of words (Condition WB). Since sentence lengths are also quite different (Simple has 17.0 words/sentence on average, Main has 25.2), the standard practice of computational linguistics of counting punctuation marks as full word tokens may also be seen as problematic. Therefore, we created two further conditions, CN (character-balanced but no punctuation) and WN (word-balanced no punctuation). In both conditions, we used the standard (Koehn, see Text S2) tokenizer to find the words, but in the N conditions we removed the punctuation chars,.?();”!:. Another potential issue concerns stemming, whether we consider the tokens *amazing, amazed, amazes* as belonging to the same or different types. To see whether this makes any difference, we also created conditions CBP, WBP, CNP, and WNP by stemming both Simple and Main using the standard Porter stemmer [38]. Table 2 compares for Simple and Main a classic measure of vocabulary richness, Herdan’s *C*, defined as log(#types)/log(#tokens), under these conditions.

**Table 2:** Vocabulary richness in Main and Simple.

Cond	SR	*C_M_*	*C_S_*	*C_M_/C_S_*
CB	1.0002	0.8226	0.8167	1.0072
CN	0.9997	0.7782	0.7739	1.0055
WB	1.0000	0.8218	0.8167	1.0061
WN	1.0000	0.7774	0.7739	1.0045
CBP	1.0002	0.8061	0.8013	1.0059
CNP	0.9997	0.7568	0.7542	1.0034
WBP	1.0000	0.8052	0.8013	1.0049
WNP	1.0000	0.7563	0.7543	1.0028

For the definition of conditions (character- or word-balanced, with or without puctuation, with or without Porter stemming) see the Methods section. SR is size ratio (number of characters in C conditions, number of words in W conditions) for comparable Main and Simple corpora. *C_M_* and *C_S_* are Herdan’s *C* for Main and Simple. As the last column shows, the vocabulary richness of comparable Simle and Main corpora differs at most by 0.72% depending on condition.

For word and part of speech (POS) n-gram statistics not all these conditions make sense, since automatic POS taggers crucially rely on information in the affixes that would be destroyed by stemming, and for the automatic detection of sentence boundaries punctuation is required [39]. We therefore used word-balanced samples with punctuation kept in place (condition WB) but distinguished different conditions of POS tagging for the following reason. Wikipedia, and encyclopedias in general, use an extraordinary amount of proper names (three times as much as ordinary English as measured e.g. on the Brown Corpus), many of which are multiword *named entities*. An ordinary POS tagger may not recognize that Long Island is a single named entity and could tag it as JJ NN (adjective noun) rather than as NNP NNP (proper name phrase). Therefore, we supplemented the original POS tagging (Condition O) by a named entity recognition (NER) system and rerun the POS tagging in light of the NER output (Condition N). If adjacent NNP-tagged elements are counted as a single NE phrase, we obtain the SO (shortened original) and SN (shortened NER-based) versions. Since neither word-based nor POS-based n-grams are very meaningful if they span sentence boundaries, we also created ‘postprocessed’ versions, where for odd n those n-grams where the boundary was in the middle were omitted, and the words/tags falling on the shorter side were uniformly replaced by the boundary marker both for odd and even n.

To measure text readability, we limited ourselves to the “Gunning fog index” *F*, [40], [41] which is one of the simplest and most reliable among all different recent and classic measures (see [42]–[44]). *F* is calculated as 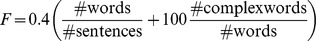
where words are considered complex if they have three or more syllables. A simple interpretation of *F* is the number of years of formal education needed to understand the text.

## Results and Discussion

We present our results in three parts. First we report on overall comparison of Main and Simple at different levels of word and n-gram statistics in addition to readability analysis. Next we narrow down the analysis further to compare selected articles and categories of articles, and examine the dependence of language complexity on the text topic. Finally, we explore the relation between controversy and language complexity by considering the case of editorial wars and related discussion pages in Wikipedia.

### Overall Comparison

#### Readability

In Table 3, the Gunning fog index calculated for 6 different English corpora is reported. Remarkably, the fog index of Simple is higher than that of Dickens, whose writing style is sophisticated but doesn’t rely on the use of longer latinate words which are hard to avoid in an encyclopedia. The British National Corpus, which is a reasonable approximation to what we would want to think of as ‘English in general’ is a third of the way between Simple and Main, demonstrating the accomplishments of Simple editors, who pushed Simple half as much below average complexity as the encyclopedia genre pushes Main above it.

**Table 3:** Readability of different English corpora.

Corpus	*F*	Corpus	*F*
Dickens	8.6±0.1	Simple	10.8±0.2
SJM	10.3±0.1	BNC	12.1±0.5
WSJ	10.8±0.2	Main	15.8±0.4

Gunning fog index for 6 different corpora of WSJ: *Wall Street Journal*•, *Charles Dickens’* books, SJM: San Jose Mercury News*, BNC: *British National Corpus* ^†^, Simple, and Main. •http://www.wsj.com *http://www.mercurynews.com ^†^ http://www.natcorp.ox.ac.uk.

#### Word statistics

Vocabulary richness is compared for Simple and Main in Table 2 using Herdan’s *C*, a measure that is remarkably stable across sample sizes: for example using only 95% of the word-balanced (Condition WB) samples we would obtain *C* values that differ from the ones reported here by less than 0.066% and 0.044%. For technical reasons we could not balance the samples perfectly (there is no sense in cutting in the middle of a line, let alone the middle of a word), but the size ratios (column SR in Table 2) were kept within 0.03%, two orders of magnitude less discrepancy than the 5% we used above, making the error introduced by less than perfect balancing negligible.

The precise choice of condition has a significant impact on *C*, ranging from a low of 0.754 (character-balanced, no punctuation, Porter stemming) to a high of 0.8226 (character-balanced, punctuation included, no stemming), but practically no effect on the 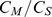 ratio, which is between 0.28% and 0.72% for all conditions reported here. In other words, we observe the same vocabulary richness in balanced samples of Simple and Main quite independent of the specific processing and balancing steps taken. We also experimented with several other tokenizers and stemmers, as well as inclusion or exclusion of numerals or words with foreign (not ISO-8859-1) characters, but the precise choice of condition made little difference in that the discrepancy between 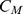 and 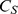 always stayed less than 1% (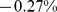 to 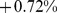). The only condition where a more significant difference of 3.4% could be observed was when Simple was directly paired with Main by selecting, wherever possible, the corresponding Main version of every Simple article.

**Figure 1:**
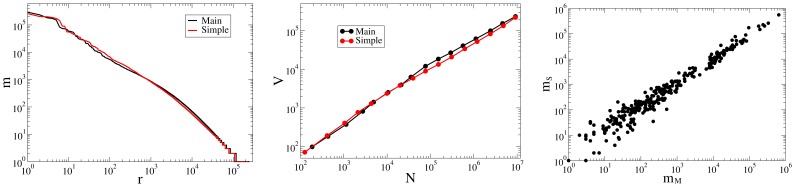
Word-level statistical analysis of Main and Simple. Condition WB, as explained the Methods section. *left:* Zipf’s law for the Main (black) and Simple (red) samples. *middle:* Heaps’ law (same colors). The exponents are 0.72±0.01 (Main) and 0.69±0.01 (Simple). *right:* Comparing token frequencies in the two samples for 300 randomly selected words (“S” and “M” stand for Simple and Main respectively), the correlation coefficient is C = 0.985. All three diagrams show that the two samples have statistically almost the same vocabulary richness.

As discussed in [45], one cannot reasonably expect the same result to hold for other traditional measures of vocabulary richness such as type-token ratio, since these are not independent of sample size asymptotically [46]. However, Herdan’s Law (also known as Heaps’ Law, [47], [48]), which states that the number of different types *V* scales with the number of tokens *N* as 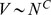, is known to be asymptotically true for any distribution following Zipf’s law [49], see [50]–[52]. In Fig. 1 (left and middle panels) our study of both laws in Condition WB, are illustrated.

Since all these results demonstrate the similarity of the Simple and Main samples in the sense of unigram vocabulary richness, a conclusion that is quite contrary to the Simple Wikipedia stylistic guidelines [53], we performed some additional tests. First, we selected 300 words randomly and compared the number of their appearance in both samples (right panel of Fig. 1). Next, we considered the word entropy of Simple and Main, obtaining 10.2 and 10.6 bits respectively. Again, the exact numbers depend on the details of preprocessing, but the difference is in the 2.9% to 3.9% range in favor of Main in every condition, while the dependence on condition is in the 1.8% to 2.8% range. Though 0.4 bits are above the noise level, the numbers should be compared to the word entropy of mixed text, 9.8 bits, as measured on the Brown Corpus, and of spoken conversation, 7.8 bits, as measured on the Switchboard Corpus. When a switch in genre can bring over 30% decrease in word entropy, a 3% difference pales in comparison. Altogether, both Simple and Main are close in word entropy to high quality newspaper prose such as the Wall Street Journal, 10.3 bits, and the San Jose Mercury News, 11.1 bits.

#### Word n-gram statistics

**Figure 2:**
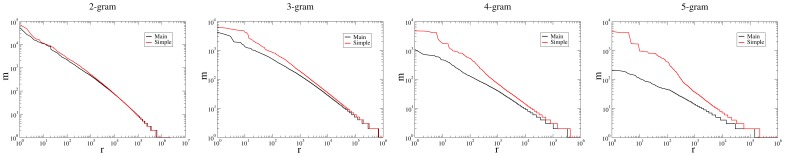
N-gram statistical analysis of Main and Simple. Condition WB, as explained the Methods section. Number of appearances of n-grams in Main (black) and Simple (red) for *n* = 2−5 from left to right. By increasing *n*, the difference between two samples becomes more significant. In Simple there are more of the frequently appearing n-grams than in Main.

One effect not measured by the standard unigram techniques is the contribution of lexemes composed of more than one word, including idiomatic expressions like ‘take somebody to task’ and collocations like ‘heavy drinker’. The Simple Wikipedia guidelines [53] explicitly warn against the use of idioms: ‘Do not use idioms (one or more words that together mean something other than what they say) ’. One could assume that Simple editors rely more on such multiword patterns, and the n-gram analysis presented here supports this. In Fig. 2 made under condition WB, the token frequencies of n-grams are shown in a Zipf-style plot as a function of their rank. Both the unigram statistics discussed in the previous section and the 2-gram statistics presented here are nearly identical for Simple and Main, but 3-grams and higher n-grams begin to show some discrepancy between them. In reality, a sample of this small size (below 10^7^ words) is too small to represent higher n-grams well, as is clear from manual inspection of the top 5-grams of Simple.

Ignoring 5-grams composed of Chinese characters (which are mapped into the same string by the tokenizer), the top four entries, with over 4200 occurrences, all come from the string. **It is found in the region**. In fact, by grepping on high frequency n-grams such as *is a commune of* we find over six thousand entries in Simple such as the following: *Alairac is a commune of 1,034 people (1999). It is located in the region Languedoc-Roussillon in the Aude department in the south of France.* Since most of these entries came from only a handful of editors, we can be reasonably certain that they were generated from geographic databases (gazetteers) using a simple ‘American Chinese Menu’ substitution tool [54], perhaps implemented as Wikipedia robots.

Since an estimated 12.3% of the articles in Simple fit these patterns, it is no surprise that they contribute somewhat to the apparent n-gram simplicity of Simple. Indeed, the entropy differential between Main and Simple, which is 0.39 bits absolute (1.7% relative) for 5-grams, decreases to 0.28 bits (1.2% relative) if these articles are removed from Simple and the Main sample is decreased to match. (By word count the robot-generated material is less than 2% of Simple, so the adjustment has little impact.) Since higher n-grams are seriously undersampled (generally, 10^9^ words ‘gigaword corpora’ are considered necessary for word trigrams, while our entire samples are below 10^7^ words) we cannot pursue the matter of multiword patterns further, but note that the boundary between the machine-generated and the manually written is increasingly blurred.

Consider *Joyeuse is a commune in the French department of Ardèche in the region of Rhône-Alpes. It is the seat of the canton of Joyeuse*, an article that clearly started its history by semi-automatic or fully automatic generation. By now (August 2012) the article is twice as long (either by manual writing or semi-automatic import from the main English wikipedia), and its content is clearly beyond what any gazetteer would list. With high quality robotic generation, editors will simply not know, or care, whether they are working on a page that originally comes from a robot. Therefore, in what follows we consider Simple in its entirety, especially as the part of speech (POS) statistics that we now turn to are not particularly impacted by robotic generation.

#### Part of speech statistics

**Figure 3:**
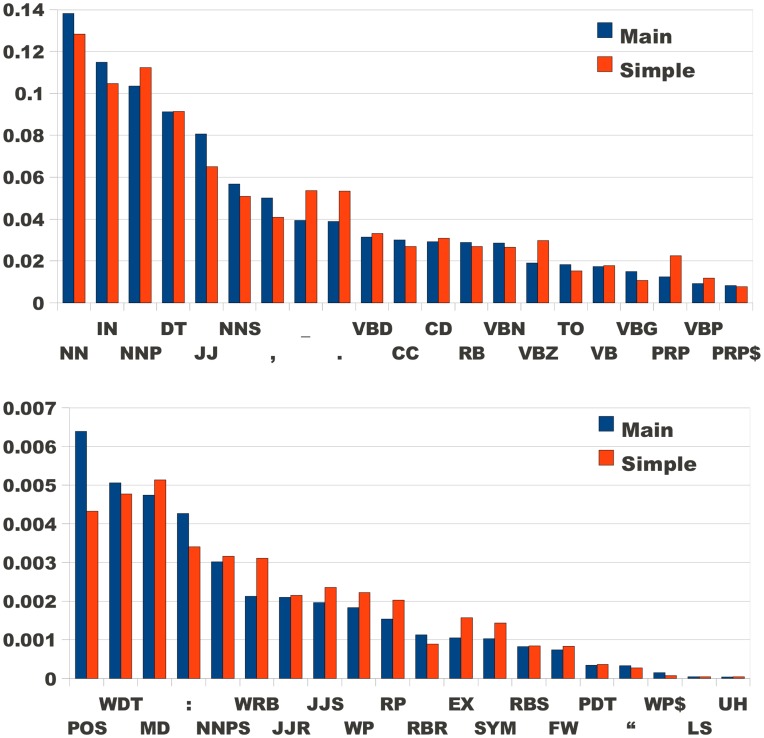
Part of Speech statistics of Main English and Simple English Wikipedias. Condition O, as explained the Methods section. The legends are defined as NN: Noun, singular or mass; IN: Preposition or subordinating conjunction; NNP: Proper noun, singular; DT: Determiner; JJ: Adjective; NNS: Noun, plural; VBD: Verb, past tense; CC: Coordinating conjunction; CD: Cardinal number; RB: Adverb; VBN: Verb, past participle; VBZ: Verb, 3rd person singular present; TO: to; VB: Verb, base form; VBG: Verb, gerund or present participle; PRP: Personal pronoun; VBP: Verb, non-3rd person singular present; PRP$: Possessive pronoun; POS: Possessive ending; WDT: Wh-determiner; MD: Modal; NNPS: Proper noun, plural; WRB: Wh-adverb; JJR: Adjective, comparative; JJS: Adjective, superlative; WP: Wh-pronoun; RP: Particle; RBR: Adverb, comparative; EX: Existential there; SYM: Symbol; RBS: Adverb, superlative; FW: Foreign word; PDT: Predeterminer; WP$: Possessive wh-pronoun; LS: List item marker; UH: Interjection.

Figure 3 shows the distribution of the part of speech (POS) tags in Main and Simple for Condition O (word balanced, punctuation and possessive *‘s* counted as separate words, as standard with the the Penn Treebank POS set [55].) It is evident from comparing the first and second columns that the encyclopedia genre is particularly heavy on Named Entities (proper nouns or phrases designating specific places, people, and organizations [56]). Since multiword entities like *Long Island, Benjamin Franklin, National Academy of Sciences* are quite common, we also preprocessed the data using the HunNER Named Entity Recognizer [57], and performed the part of speech tagging afterwards (condition N). When adjacent NNP words are counted as one, we obtained the SO and SN conditions. This obviously affects not just the NNP counts, but also the higher n-grams that contain NNP.

Again, the similarity of Simple and Main is quite striking: the cosine similarity measure of these distributions is between 0.989 (Condition O) and 0.991 (Condition SO), corresponding to an angle of 7.7 to 8.6 degrees. To put these numbers in perspective, note that the similarity between Main and the Brown Corpus is 0.901 (25.8 degrees), and between Main and Switchboard 0.671 (47.8 degrees). For POS n-grams, it makes sense to omit n-grams with a sentence boundary at the center. For the POS unigram models this means that we do not count the notably different sentence lengths twice, a step that would bring cosine similarity between Simple and Main to 0.992 (Condition SO) or 0.993 (Condition N), corresponding to an angle of 6.8 to 7.1 degrees. Either way, the angle between Simple and Main is remarkably acute.

While Figure 3 shows some slight stylistic variation, e.g. that Simple uses twice as many personal pronouns (*he, she, it, …*) as Main, it is hard to reach any overarching generalizations about these, both because most of the differences are statistically insignificant, and because they point in different directions. One may be tempted to consider the use of pronouns to be an indicator of simpler, more direct, and more personal language, but by the same token one would have to consider the use of wh-adverbs (*how however whence whenever where whereby wherever wherein whereof why …*) to be a hallmark of more sophisticated, more logical, and more impersonal style, yet it is Simple that has 50% more of these.

**Figure 4.POS-N-gram:**
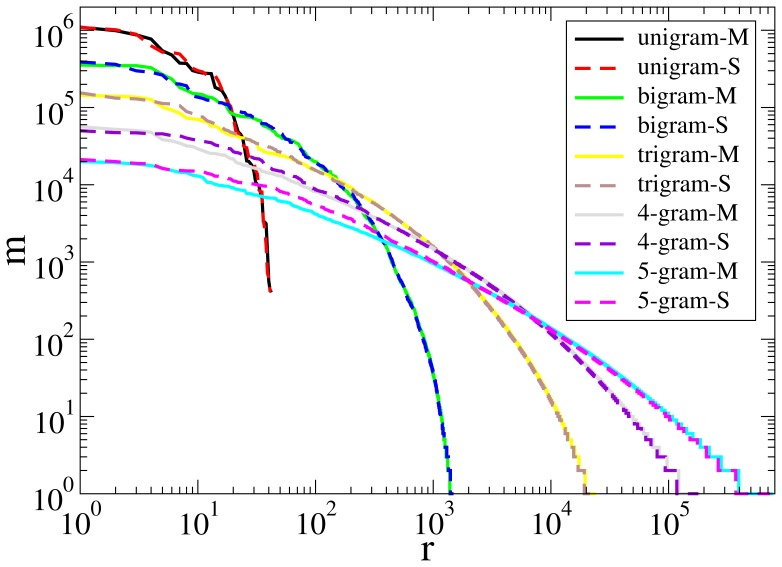
N-gram statistical analysis of Main and Simple Number of appearances of POS n-grams in Main and Simple for *n* = 1–5 under condition N.

Figure 4 shows that the POS n-gram Zipf plots for 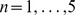 are practically indistinguishable across Simple and Main under Condition N. (We are publishing this figure as it is the worst – under the other conditions, the match is even better.) In terms of cosine similarity, the same tendencies that we established for unigram data remain true for bigram or higher POS n-grams: the Switchboard data is quite far from both Simple and Main, the Brown Corpus is closer, and the WSJ is closest. However, Simple and Main are noticeably closer to one another than either of them is to WSJ, as is evident from the Table 4, which gives the angle, in decimal degrees, between Simple and Main (column SM), Main and WSJ (column MW), and Simple and WSJ (column SW) based on POS n-grams for 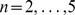, under condition SN, with postprocessing of n-grams spanning sentence boundaries. We chose this condition because we believe it to be the least noisy, but we emphasize that the same relations are observed for all other conditions, with or without sentence boundary postprocessing, with or without removal of machine-generated entries from Simple, with or without readjusting the Main corpus to reflect this change (all 32 combinations were investigated). The data leave no doubt that the WSJ is closer to Main than to Simple, but the angles are large enough, especially when compared to the Simple/Main column, to discourage any attempt at explaining the syntax of Main, or Simple, based on the syntax of well-edited journalistic prose. We conclude that the simplicity of Simple, evident both from reading the material and from the Gunning Fog index discussed above, is due primarily to Main having considerably longer sentences. A secondary effect may be the use of shorter subsentences (comma-separated stretches) as well, but this remains unclear in that the number of subsentence separators (commas, colons, semicolons, parens, quotation marks) per sentence is considerably higher in Main (1.62) than in Simple (1.01), so a Main subsentence is on the average not much longer than a Simple subsentence (8.62 vs 7.96 content words/subsentence).

**Table 4:** Statistical similarity between different samples at different length of n-grams.

n	SM	MW	SW
2	13.1	28.3	33.8
3	16.5	33.4	40.4
4	20.1	40.8	49.8
5	28.7	47.9	58.2

Angle, in decimal degrees, between Simple and Main (column SM), Main and WSJ (column MW), and Simple and WSJ (column SW) based on POS n-grams for 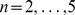, under condition SN, with postprocessing of n-grams spanning sentence boundaries.

### Topical Comparison

Clearly, readability of text is a very context dependent feature. The more conceptually complex a topic, the more complex linguistic structures and the less readability are expected. To examine this intuitive hypothesis, we considered different articles in different topical categories. Instead of systematically covering all possible categories of articles, here we illustrate the phenomenon on a limited number of cases, where significant differences are observed. The readability index of 10 selected articles from different topical categories is measured and reported in in Table 5.

**Table 5:** Comparison of readability in Main and Simple English Wikipedias.

Article	*F*_M*ain*_	*F*_S*imple*_
Philosophy	16.6	11.3
Physics	15.9	11.1
Politics	14.1	8.9
You’re My Heart, You’re My Soul (song)	9.6	5.8
Real Madrid C.F.	11.6	7.6
Immanuel Kant	15.7	10.3
Albert Einstein	13.5	8.9
Barack Obama	12.7	9.7
Madonna (entertainer)	11.2	8.9
Lionel Messi	12.8	7.9

Gunning fog index for the same example articles in Main and Simple.

While these results are clearly indicative of the main tendencies, for more reliable statistics we need larger samples. To this end we sampled over 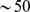 articles from 10 different categories and averaged the readability index for the articles within the category. Results are shown in Table 6. The numbers make it clear that more sophisticated topics, e.g. *Philosophy* and *Physics* require more elaborate language compared to the more common topics of *Politics* and *Sport*. In addition, there is considerable difference between subjective and objective articles, in that the level of complexity is slightly higher in the former: more objective articles (e.g. biographies) are more readable.

**Table 6:** Readability in different topical categories.

Category	*F*_M*ain*_	*F*_S*imple*_
Philosophy	17.2±0.6	12.7±0.8
Physics	16.5±0.4	11.3±0.7
Politics	14.0±0.5	11.2±0.8
Songs	13.3±0.6	11.0±0.7
Sport clubs	12.2±0.7	10.1±0.6
Philosophers	15.9±0.6	11.5±0.8
Physicists	15.0±0.5	10.0±0.7
Politicians	13.1±0.4	10.2±0.6
Singers	13.2±0.4	10.1±0.5
Athletes	13.1±0.3	10.1±0.6

Gunning fog index for samples of articles in 10 different categories in Main and Simple.

### Conflict and Controversy

Wikipedia pages usually evolve in a smooth, constructive manner, but sometimes severe conflicts, so called *edit wars*, emerge. A measure *M* of controversially was coined by appropriately weighting the number of mutual reverts with the number of edits of the participants of the conflict in our previous works [18], [58], [59]. (For the exact definition and more details, see Text S1.) By measuring *M* for articles, one could rank them according to controversiality (the intensity of editorial wars on the article).

In order to enhance the collaboration, resolve the issues, and discuss the quality of the articles, editors communicate to each other through the “talk pages” [60] both in controversial and in peacefully evolving articles. Depending on the controversially of the topic, the language that is used by editors for these communications can become rather offensive and destructive.

In classical cognitive sociology [61], there is a distinction between “constructive” and “destructive” conflicts. “Destructive processes form a coherent system aimed at inflicting psychological, material or physical damage on the opponent, while constructive processes form a coherent system aimed at achieving one’s goals while maintaining or enhancing relations with the opponent” [62]. There are many characteristics that distinguish these two types of interactions, such as the use of swearwords and taboo expressions, but for our purposes the most important is the lowering of language complexity in the case of destructive conflict [62].

Since we can locate destructive conflicts in Wikipedia based on measuring *M*, a computation that does not take linguistic factors into account, we can check independently whether linguistic complexity is indeed decreased as the destructivity of the conflict increases. To this end, we created two similarly sized samples, one composed of 20 highly controversial articles like *Anarchism* and *Jesus*, the other composed of 20 peacefully developing articles like *Deer* and *York*. The Gunning fog index was calculated both for the articles and the corresponding talk pages for both samples. Results are shown in Table 7. We see that the fog index of the conflict pages is significantly higher than those of the peaceful ones (with 99.9% confidence calculated with Welch’s t-test). This is in accord with the previous conclusion about the topical origin of differences in the index (see Table 6): clearly, conflict pages are usually about rather complex issues.

**Table 7:** Controversy and readability.

	Controversial	Peaceful
*F*_A*rticle*_	16.5±0.9	11.6±0.4
*F*_T*alk*_	11.7±0.6	8.6±0.8
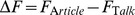	4.8	3.0

Gunning fog index for two sample articles of highly controversial and peaceful articles and the corresponding talk pages.

In both samples there is a notable decrease in the fog index when going from the main page to the talk page, but this decrease is considerably larger for the conflict pages (4.8 vs. 3.0, separated within a confidence interval of 85%). This is just as expected from earlier observations of linguistic behavior during destructive conflict [62]. The language complexities for controversial articles and the corresponding talk pages are higher to begin with, but the amount of reduction in language complexity 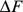 is much more noticeable in the presence of destructive conflicts and severe editorial wars.

### Conclusions and Future Work

In this work we exploited the unique near-parallelism that obtains between the Main and the Simple English Wikipedias to study empirically the linguistic differences triggered by a single stylistic factor, the effort of the editors to make Simple simple. We have found, quite contrary to naive expectations, and to Simple Wikipedia guidelines, that classic measures of vocabulary richness and syntactic complexity are barely affected by the simplification effort. The real impact of this effort is seen in the less frequent use of more complex words, and in the use of shorter sentences, both directly contributing to a decreased Fog index.

Simplification of the lexicon, as measured by *C* or word entropy, is hardly detectable, unless we directly compare the corresponding Simple and Main articles, and even there the effect is small, 3.4%. The amount of syntactic variety, as measured by POS n-gram entropy, is decreased from Main to Simple by a more detectable, but still rather small amount, 2–3%, with an estimated 20–30% of this decrease due to robotic generation of pages. Altogether, the complexity of Simple remains quite close to that of newspaper text, and very far from the easily detectable simplification seen in spoken language.

We believe our work can help future editors of the simple Wikipedia, e.g. by adding robotic complexity checkers. Further investigation of the linguistic properties of Wikipedias in general and the simple English edition in particular could provide results of great practical utility not only in natural language processing and applied linguistics, but also in foreign language education and improvement of teaching methods. The methods used here may also find an application in the study of other purportedly simpler language varieties such as creoles and child-direceted speech.

## Supporting Information

Text S1 **Controversy measure.** Detailed description and definition of controversy measure *M*.
(PDF)

Text S2 **Corpora and analysis tools.** Detailed protocol of text-mining process and directions to the software and corpora.
(PDF)
