# Supplementary material for: A Practical Approach to Language Complexity: A Wikipedia Case Study
Source: PLoS One. 2012 Nov 7;7(11):e48386. doi: 10.1371/journal.pone.0048386 (PMC3492358; doi:10.1371/journal.pone.0048386)
Supplement: Text S1 — Controversy measure. Detailed description and definition of controversy measure M. (PDF) [file pone.0048386.s001.pdf]

Text S1: Controversy measure

To quantify the controversiality of an article based on its editorial history, we focus on "reverts", i.e. when an editor undoes another editor's edit completely. To detect reverts, we first assign a MD5 hash code [1] to each revision of the article and then by comparing the hash codes, detect when two versions in the history line are exactly the same. In this case, the latest edit (leading to the second identical revision) is marked as a revert, and a pair of editors, namely a reverting and a reverted one, are recognized. A "mutual revert" is recognized if a pair of editors (x, y) is observed once with x and once with y as the reverter. The weight of an editor x is defined as the number of edits N performed by her, and the weight of a mutually reverting pair is defined as the minimum of the weights of the two editors. The controversiality M of an article is defined by summing the weights of all mutually reverting editor pairs, excluding the topmost pair, and multiplying this number by the total number of editors E involved in the article. In formula,

$$M = E \sum_{\text{all mutual reverts}} min(N^{\mathrm{d}}, N^{\mathrm{r}}), \tag{1}$$

where $N^{\mathrm{r/d}}$ is the number of edits on the article committed by reverting/reverted editor. The sum is taken over mutual reverts rather than single reverts because reverting is very much part of the normal workflow, especially for defending articles from vandalism. The minimum of the two weights is used because conflicts between two senior editors contributing more to controversiality than conflicts between a junior and a senior editor, or between two junior editors. For more details on how the above formula defining M was selected and validated see [2] and especially *Text S1* in its Supporting Information.

References

1. Rivest RL (1992) The md5 message-digest algorithm. Internet Request for Comments : RFC 1321.

2. Yasseri T, Sumi R, Rung A, Kornai A, Kertész J (2012) Dynamics of conflicts in Wikipedia. PLoS ONE 7: e38869.
