# Supplementary material for: A Practical Approach to Language Complexity: A Wikipedia Case Study
Source: PLoS One. 2012 Nov 7;7(11):e48386. doi: 10.1371/journal.pone.0048386 (PMC3492358; doi:10.1371/journal.pone.0048386)
Supplement: Text S2 — Corpora and analysis tools. Detailed protocol of text-mining process and directions to the software and corpora. (PDF) [file pone.0048386.s002.pdf]

Text S2: Corpora and analysis tools

To download Wikipedia dumps use the static snapshots from http://dumps.wikimedia.org. To download the dynamic content, especially the most updated version of individual articles, use the "MediaWiki API" online platform accessible at http://www.mediawiki.org/wiki/API:Main_page. The Brown, Switchboard, and WSJ corpora are distributed by the Linguistic Data Consortium as part of the Penn Treebank, http://www.ldc.upenn.edu/Catalog/catalogEntry.jsp?catalogId=LDC99T42 The POS tagging of these texts, while not necessarily 100% correct, is manually corrected and generally considered a gold standard against which POS taggers are evaluated. Many gigaword corpora (including Arabic, Chinese, English, French, and Spanish) are available from the LDC, see http://www.ldc.upenn.edu/Catalog/catalogSearch.jsp

To clean the text from Wikimedia tags and external references, we used the WikiExtractor developed at the University of Pisa Multimedia Lab, available at http://medialab.di.unipi.it/wiki/Wikipedia_Extractor. Another system with similar capabilities is "wiki2text" http://wiki2text.sourceforge.net. We used faster (flex-based) versions of the original Koehn tokenizer and Mikheev sentence splitter, available at https://github.com/zseder/webcorpus.

For English stemming, the standard is the "Porter Stemming Algorithm" http://tartarus.org/~martin/PorterStemmer. For other languages a good starting point is http://aclweb.org/aclwiki/index.php?title=List_of_resources_by_language.

We calculated the Gunning Fog index using the code and algorithm of Greg Fast http://cpansearch.perl.org/src/GREGFAST/Lingua-EN-Syllable-0.251/Syllable.pm. For part-of-speech tagging we used the "HunPOS tagger" http://code.google.com/p/hunpos/ and the "HunNER NE recognizer", which are specific applications of the "HunTag tool", available at https://github.com/recski/HunTag/.

To perform the n-gram analysis we used the "N-Gram Extraction Tools" http://homepages.inf.ed.ac.uk/lzhang10/ngram.html of Le Zhang.

All the abovementioned code and packages are open source and available publicly under GPL, LGPL, or similar licenses, but some corpora may have copyright restrictions.
